# Supplementary material for: Secondary teachers’ competencies and attitude: A mediated multigroup model based on usefulness and enjoyment to examine the differences between key dimensions of STEM teaching practice
Source: PLoS One. 2023 Jan 26;18(1):e0279986. doi: 10.1371/journal.pone.0279986 (PMC9879531; doi:10.1371/journal.pone.0279986)
Supplement: S1 Table — (DOCX) [file pone.0279986.s001.docx]

**Supplementary Table S1****. Survey questionnaire.**

| **Each teacher is requested to weight the following variables on a seven-point Likert scale** (*(1) Total disagreement (2) Disagree (3) Disagree in some extent (4) Neutral (5) Agree in some extent (6) Agree (7) Totally agree*) **to explore in which contexts the teachers' competency is a key predictor of attitude regarding Inquiry-Based Learning (IBL) and Integration of INT content (INT) INT** | |
| --- | --- |
| **Competences** *(adopted by [1])* | |
| SI12. I can identify topics appropriate for teaching through IBL/INT to help students better understand the problematic content. |  |
|  |  |
| SI13. I can identify topics suitable for teaching through IBL/INT to increase the effectiveness of the teaching process. |  |
|  |  |
| SI14. Through IBL/INT I can adapt the lesson content to the student's understanding. |  |
|  |  |
| SI15. I can use IBL/INT to develop the learners' creativity. |  |
|  |  |
| SI16. I can use IBL/INT to stimulate learners to observe, explore and investigate. |  |
|  |  |
| SI17. I can identify appropriate strategies such as IBL/INT to stimulate students' collaboration. |  |
|  |  |
| SI18. I can identify appropriate strategies such as IBL/INT for integrating technology into the classroom to stimulate students' collaboration. |  |
|  |  |
| SI19. I can identify appropriate strategies such as IBL/INT for integrating technology into the classroom to stimulate students' critical thinking. |  |
|  |  |
| **Attitude** *(adopted by [2, 3])* | |
| SI.31. INT/IBL strategy is diverse and has various paths. |  |
|  |  |
| SI32. INT/IBL facilitates exploring and understanding some real-life situations. |  |
|  |  |
| SI33. IBL/INT implementation stages are according to the scientific rigors. |  |
|  |  |
| SI34. IBL/INT leads to the stimulation of learners' autonomy. |  |
|  |  |
| SI35. IBL/INT leads to the stimulation of a critical analysis of experimental data. |  |
|  |  |
| SI36. IBL/INT provides learners with a learning algorithm. |  |
|  |  |
| SI37. IBL/INT enhances learners' curiosity. |  |
|  |  |
| SI38. IBL/INT stimulates teamwork. |  |
|  |  |
| SI39. The teacher has a facilitating role during the learning process using IBL/INT |  |
|  |  |
| SI310. Using IBL/INT The learner is actively involved in the learning process if IBL/INT is used. |  |
|  |  |
| **Enjoyment** | |
| SI53. IBL/INT activities are boring. (R) |  |
|  |  |
| SI54. IBL/INT activity never attracted my attention. (R) |  |
|  |  |
| SI55. IBL/INT activity is very interesting. |  |
|  |  |
| SI56. IBL/INT activity is an enjoyable experience. |  |
|  |  |
| **Usefulness** | |
| SI63. Because it seems useful, I would put a lot of efforts to promote IBL/INT activities. |  |
|  |  |
| S164. Because it seems useful, I would put a lot of efforts to implement IBL/INT activities. |  |
|  |  |
| SI71. I would be inclined to apply IBL/INT, because I find it a valuable strategy for all educational actors. |  |
|  |  |
| SI72. I would be inclined to apply IBL/INT because I find it useful for teaching act |  |
|  |  |

*Note: R - Reverse item*

1. Chuang H-H, Weng C-Y, Huang F-C. A structure equation model among factors of teachers' technology integration practice and their TPCK. Computers & Education. 2015;86:182-91.

2. Thibaut L, Knipprath H, Dehaene W, Depaepe F. How school context and personal factors relate to teachers' attitudes toward teaching integrated STEM. International Journal of Technology and Design Education. 2018;28. doi: 10.1007/s10798-017-9416-1.

3. Thibaut L, Knipprath H, Dehaene W, Depaepe F. The influence of teachers' attitudes and school context on instructional practices in integrated STEM education. Teaching and Teacher Education. 2018;71:190-205.
