# Supplementary material for: Secondary teachers’ competencies and attitude: A mediated multigroup model based on usefulness and enjoyment to examine the differences between key dimensions of STEM teaching practice
Source: PLoS One. 2023 Jan 26;18(1):e0279986. doi: 10.1371/journal.pone.0279986 (PMC9879531; doi:10.1371/journal.pone.0279986)
Supplement: Attachment — Submitted filename: Response to Reviewer 2.pdf [file pone.0279986.s004.pdf]

Dear Reviewer,

Sincerest thanks for your valuable time and useful contribution.

We incorporated all your suggestions, and we are grateful to you for careful reading of the paper and helpful suggestions and comments to improve our work.

We provide our responses point by point and modify the manuscript according to the accurate and intuitive comments.

Reviewer #2:

This study is coherent and rigorous, both in terms of theoretical underpinning and methodology. It adequately sets out the research objectives and study hypothesis. The data analysis processes are robust and in line with the research objectives. Results are well described and respond to the research questions. Conclusions are appropriate.

Authors should review the bibliography, as they do not use the "Vancouver" style, as indicated in the journal's guidelines.

R: The authors agree that were omissions in the bibliography. Consequently, the bibliography was improved.

We hope you find this revision appropriate for research topic. Once more, thank you for your exhaustive review.

Sincerely,

Dr. Iulia Lazar, corresponding author

On behalf of all authors
