# Supplementary material for: Secondary teachers’ competencies and attitude: A mediated multigroup model based on usefulness and enjoyment to examine the differences between key dimensions of STEM teaching practice
Source: PLoS One. 2023 Jan 26;18(1):e0279986. doi: 10.1371/journal.pone.0279986 (PMC9879531; doi:10.1371/journal.pone.0279986)
Supplement: Attachment — Submitted filename: Response to Reviewer 1_revision 2.docx [file pone.0279986.s005.docx]

Dear Reviewer,

Sincerest thanks for your valuable time and valuable contribution.

We incorporated all your suggestions, and we are grateful to you for your careful reading of the paper and helpful advice and comments to improve our work.

We provide our responses point by point and modify the manuscript according to accurate and intuitive comments.

Due to significant changes in some parts of the manuscript, some errors cannot be corrected in the change mode (‘track changes’). In addition, we mention that the lines indicated in the responses to the reviewer correspond to the corrected final version without track changes. However, some differences can be observed between the “clean” and ~track changes~form.

All authors agree with the content of the manuscript after the second revision. Also, I confirm that co-authors all had an active part in the final manuscript.

As the corresponding author, I take responsibility for informing co-authors on time of editorial decisions, received reviews, changes made in response to the editorial review, and the content of revisions.

**In addition, we mention that the lines indicated in the responses to the reviewer *correspond to the corrected final version without track changes*.**

Reviewer #1:

1. Three hundred teachers (i.e., mathematics, physics, chemistry, biology, computing, ICT, 330 Technologies) who used IBL and INT teaching practices have been interviewed. How to make sure 300 teachers are interviewed? How to be conducted?

2. How to get the number of 300 sample from the population? What sampling method used?

*R to Question 1 and Question 2: The participants and data collection procedure section has been rewritten (see Lines 325-369).*

3. Based on demographic, the specialization of teachers are not balances. How can this study concluded that all the teachers can be generalized to the population number based on specialization?

*R: Based on supplementary searching, we reorganized and extended the Limitations and suggestions for future research section (see Lines 588-602).*

4. Show the results of effect size

*R: The effect size results were included (see Lines* *489-500*).

5. Discussion should be referred to the finding. Discuss the significant indirect effects for total sample (a), IBL (b) and INT (c) teaching practices. Discuss with pro and contra. Add citations to support the statement.

*R: We extended the discussion part of this study (see Lines 547-555)*

7. PLOS authors have the option to publish the peer review history of their article (what does this mean?). If published, this will include your full peer review and any attached files.

*R: Yes, we agree with the option to publish the peer review history of their article.*

Reviewers’ comments:

3. Has the statistical analysis been performed appropriately and rigorously?

Reviewer #1: No

*R: Measurement invariance composite (MICOM) results based on three steps (configural invariance, compositional invariance, & equality of composite mean values and variances) revealed that full measurement invariance has been established. This analysis was only briefly detailed because of the complexity and the already considerable size of the article. However, supplementary material was presented. (see Lines 438-446).*

Sincerely,

Dr. Iulia Lazar, corresponding author

On behalf of all authors
