# Supplementary material for: Secondary teachers’ competencies and attitude: A mediated multigroup model based on usefulness and enjoyment to examine the differences between key dimensions of STEM teaching practice
Source: PLoS One. 2023 Jan 26;18(1):e0279986. doi: 10.1371/journal.pone.0279986 (PMC9879531; doi:10.1371/journal.pone.0279986)
Supplement: Attachment — Submitted filename: Response to Reviewer 1_revision 3.docx [file pone.0279986.s006.docx]

Dear Reviewer,

Sincerest thanks for your valuable time and valuable contribution.

We incorporated all your suggestions, and we are grateful to you for your careful reading of the paper and helpful advice and comments to improve our work.

We provide our responses point by point and modify the manuscript according to accurate and intuitive comments.

Due to significant changes in some parts of the manuscript, some errors cannot be corrected in the change mode (‘track changes’). In addition, we mention that the lines indicated in the responses to the reviewer correspond to the corrected final version without track changes. However, some differences can be observed between the “clean” and ~track changes~ forms.

All authors agree with the content of the manuscript after the second revision. Also, I confirm that co-authors all had an active part in the final manuscript.

As the corresponding author, I take responsibility for informing co-authors on time of editorial decisions, received reviews, changes made in response to the editorial review, and the content of revisions.

**In addition, we mention that the lines indicated in the responses to the reviewer *correspond to the corrected final version without track changes*.**

Reviewer #1:

1. Method :
- Elaborate research design
- Why using snowball, it was for qualitative research
- Sub-topic on method should be include :

- Research design,
- Participant,
- Measurement,
- Data analysis,
- EFA test result should be elaborate **in part of method** (KMO and berlette, Communalities, eigenvalue, % cumulative of variance, rotation matrics)

- EFA result should be elaborate. Make sure all of this ellaborate.

*R to Question 1 and Question 2: The method section has been partially rewritten (see the manuscript version with track changes).*

3. Results

- Elaborate based hypothesis listed

- Elaborate the assumption using SEM by smartPLS: normality test, multicollinearity, discriminant etc

*R: We reorganized and extended the result section (see the manuscript version with track changes).*

4. Discussion

- Discuss the finding based hypothesis listed

- Use idea, justification and reason to discuss research finding

- elaborate the contribution of this study.

*R: We reorganized and extended the discussion section (see the manuscript version with track changes).*

*We would also like to express our gratitude for all your consistent suggestions, in the absence of which we wouldn’t have succeeded in rigorously partially rewriting the manuscript.*

Sincerely,

Dr. Iulia Lazar, corresponding author

On behalf of all authors
