# Supplementary material for: Secondary teachers’ competencies and attitude: A mediated multigroup model based on usefulness and enjoyment to examine the differences between key dimensions of STEM teaching practice
Source: PLoS One. 2023 Jan 26;18(1):e0279986. doi: 10.1371/journal.pone.0279986 (PMC9879531; doi:10.1371/journal.pone.0279986)
Supplement: Attachment — Submitted filename: Response to Reviewer 1_revision 4.docx [file pone.0279986.s007.docx]

Dear Reviewer,

All authors agree with the content of the manuscript after the fourth revision. Also, I confirm that co-authors all had an active part in the final manuscript.

As the corresponding author, I take responsibility for informing co-authors on the time of editorial decisions, received reviews, changes made in response to the editorial review, and the content of revisions.

**In addition, we mention that the lines indicated in the responses to the reviewer *correspond to the corrected final version without track changes*.**

Reviewer #1:

1. Find references for communalities value less than 0.2 are accepted
*R to Suggestion 1: The Data analysis subsection has been partially rewritten after statistical corrections. Now, all communalities are larger than 0.4. See in the document “Revised 4 Manuscript”- Table 2. Factor Loadings from Exploratory Factor Analysis for total sample (N=600)*

2. Find citations for percentages of total variance less than 60% are accepted.

*R to Suggestion 2: Now, the percentages of total variance are higher than 60%. See in the document “Revised 4 Manuscript” – lines 474-482*

3. Based on experience to use EFA, this study's result was not good. Some of the items should be removed. but the author still proceeds with the item with a communalities value less than 0.4 or 0.5*.*

*R to Suggestion 3: The EFA analyses were revised. See in the document “Revised 4 Manuscript”– lines 474-487*

4. Results pilot test for CFA are not clear. Need to show the value of factor loading, correlation, AVE, Composite reliability, and Cronbach Alpha, Not only of model fit value.

*R to Suggestion 4: The CFA analyses were revised. See in the document “Revised 4 Manuscript”– lines 544-565*

5. Actual study results need to be re-analysis after checking a pilot test result.

*R to Suggestion 5: Actual study results were re-analysis after checking a pilot test result.*

*We would also like to express our gratitude for all your consistent suggestions, in the absence of which we wouldn’t have succeeded in rigorously partially rewriting the manuscript.*

Sincerely,

Dr. Iulia Lazar, corresponding author

On behalf of all authors
