# Supplementary material for: Secondary teachers’ competencies and attitude: A mediated multigroup model based on usefulness and enjoyment to examine the differences between key dimensions of STEM teaching practice
Source: PLoS One. 2023 Jan 26;18(1):e0279986. doi: 10.1371/journal.pone.0279986 (PMC9879531; doi:10.1371/journal.pone.0279986)
Supplement: Attachment — Submitted filename: PONE-D-21-14168_R4.docx [file pone.0279986.s009.docx]

This is a valuable study in which a model was developed using a partial least square structural equation modelling (PLS-SEM) approach, showing that teacher competence has direct effects on attitudes and indirect effects on usefulness and enjoyment. The results may be used to improve the effectiveness of STEM teaching.

The study used the snowball sampling method. Starting from the initial list, access to a considerable number of STEM teachers has improved. The study had a large sample size and used multiple scales. The mediated multigroup model proposed in this study contributes to the Will, Skill, Tool (WST) model.
