# Supplementary material for: Secondary teachers’ competencies and attitude: A mediated multigroup model based on usefulness and enjoyment to examine the differences between key dimensions of STEM teaching practice
Source: PLoS One. 2023 Jan 26;18(1):e0279986. doi: 10.1371/journal.pone.0279986 (PMC9879531; doi:10.1371/journal.pone.0279986)
Supplement: Attachment — Submitted filename: Reviewer comments.docx [file pone.0279986.s008.docx]

# Reviewer Comments

**Reviewer 1:**

**General remarks:**

The overall manuscript is very focused. This research explores the mediating role of perceived usefulness and enjoyment of STEM teaching practice between secondary teachers' competencies and attitudes through structural equation model. Meanwhile, the study examines whether the relationship between model structure differs for Inquiry-based learning (IBL) and STEM content integration (INT), which is an interesting and innovative finding.

However, some sentences contain redundant words. One serious issue I spot is the lack of coherence in the theoretical framework. Some of the arguments are illogical and the arguments are not thorough enough, which makes reading difficult.

**Specific remarks:**

p. abstracts. The last sentence contains a statement: “Understanding the mediating role of perceived usefulness and enjoyment for each STEM practice would help teachers implement

STEM education.” It needs to be explained in detail in your manuscript later on.

p.1 lines 44-45. References to STEAM definitions are best marked with the page number of the original text.

p.4 line 68. IBL emphasizes critical thinking and inquiry-based learning. However, in IBL mode, why is it important to explore the factors affecting learners' attitudes? This logic should be further elaborated. Additional, if the term IBL appears for the first time, it is recommended to explain.

p.5 lines 75-82, the first sentence does not have a strong logical connection with the content elaborated later. It is suggested to further demonstrate the reasons why IBL or INT method in STEM is difficult to implement.

p.10, the concept logic of teacher's ability and student's ability is not clear enough in the discussion of the three elements of ability, especially in lines 115-117. It is suggested to reorganize the sentences to make the logic more coherent.

p.13, lines 145-146, "Moreover, usefulness mediates the relationship between ease of use and attitude" suggest adding literature citation support.

p.25, lines 235-241, the logic of the argument that attitude depends on cognitive ability needs to be reorganized. What new cognitive needs does STEM teaching practice put forward for teachers' cognitive ability, which is different from other teaching environments, and there is no logical connection between the first and second.

p.27, line 253, the summary of the hypothesis can be more concise. There are similar contents in the introduction and the previous literature review, which can be carefully considered

The WST model explains the relationship between attitude and ability, but whether the other two mediating variables can be deeply explained by different model theories, for example, whether perceived usefulness and attitude can be explained by Technology Acceptance Model (TAM), and whether enjoyment can be explained by Flow Theory.

p.54, line 473, there is no Table 2A. Please check the chart and the passage carefully.

Table 3b, line 543, place parameters in italics (*p*). Also check the remainder of your manuscript for this.

In the end, the Path diagram can be refined, for example by adding a topic item.
